# 

**Published:** 1896-04

**Authors:** 


### CONTENTS.

ORIGINAL COMMUNICATIONS—

A BRIEF HISTORY OF OTOLOGY FROM ITS INFANCY TO THE PRESENT TIME. By T. E. Mitchell, M.D., Columbus, Ga. .... 73

A CASE OF URINARY INFILTRATION OF SCROTUM, MISTKEN FOR ORCHITIS. PERINEAL URETHROTOMY, CONSECUTIVE GANGRENE AND DEATH. By Thomas H. Manley, M.D., New York .... 80

A PECULIAR FORM OF IRITIS CHARACTERIZED BY A GELATINOUS MASS IN ANTERIOR CHAMBER, AT TIMES RESEMBLING A CATARACTOUS LENS. By S. Latimer Phillips, M.D., Savannah, Ga .... 84

THE SURGICAL TREATMENT OF RETRO-DEVIATIONS OF THE UTERUS. By Augustin H. Goelet, M.D., New York .... 88

REPORT OF A CASE OF ADDISON'S DISEASE. By J. N. Hall, M.D., Denver, Colorado. 90

NOTE ON THE HISTORY OF SYPHILIS. By E. Van Goidtsnoven, M.D., Atlanta, Ga... 92

SOCIETY REPORTS—

CLINICAL SOCIETY OF MARYLAND .... 94

CORRESPONDENCE—

THE FITZGERALD COLONY .... 102

GANGRENE RESULTING FROM BITE OF "BLUE-GUM" NEGRO .... 103

CASE OF EARLY PREGNANCY .... 105

EDITORIAL—

THE AMERICAN MEDICAL ASSOCIATION .... 106

AMERICAN ACADEMY OF MEDICINE .... 109

THE MEDICAL ASSOCIATION OF GEORGIA .... 112

COLLEGE COMMENCEMENTS .... 113

SELECTIONS AND ABSTRACTS—

OBSTETRICS AND GYNECOLOGY .... 117

GENERAL MEDICINE AND THERAPEUTICS .... 119

SKIN DISEASES AND SYPHILIS .... 129

SURGERY .... 131

MEDICAL ITEMS .... 134

BOOK REVIEWS .... 138

ADVERTISERS' INDEX TO ADVERTISEMENTS .... xiv

MEDICAL ITEMS—CONTINUED .... x, xi, xii, xiii, xv

CLASSIFIED INDEX TO ADVERTISEMENTS .... xxxi

